# Selenium Enrichment of Horticultural Crops

**DOI:** 10.3390/molecules22060933

**Published:** 2017-06-04

**Authors:** Martina Puccinelli, Fernando Malorgio, Beatrice Pezzarossa

**Affiliations:** 1 Department of Agriculture, Food and Environment, University of Pisa, via del Borghetto 80, 56124 Pisa, Italy; martina.puccinelli@for.unipi.it (M.P.); fernando.malorgio@unipi.it (F.M.); 2 Institute of Ecosystem Study, CNR, via Moruzzi 1, 56124 Pisa, Italy

**Keywords:** biofortification, antioxidant, plant senescence, post-harvest

## Abstract

The ability of some crops to accumulate selenium (Se) is crucial for human nutrition and health. Selenium has been identified as a cofactor of the enzyme glutathione peroxidase, which is a catalyzer in the reduction of peroxides that can damage cells and tissues, and can act as an antioxidant. Plants are the first link in the food chain, which ends with humans. Increasing the Se quantity in plant products, including leafy and fruity vegetables, and fruit crops, without exceeding the toxic threshold, is thus a good way to increase animal and human Se intake, with positive effects on long-term health. In many Se-enriched plants, most Se is in its major organic form. Given that this form is more available to humans and more efficient in increasing the selenium content than inorganic forms, the consumption of Se-enriched plants appears to be beneficial. An antioxidant effect of Se has been detected in Se-enriched vegetables and fruit crops due to an improved antioxidative status and to a reduced biosynthesis of ethylene, which is the hormone with a primary role in plant senescence and fruit ripening. This thus highlights the possible positive effect of Se in preserving a longer shelf-life and longer-lasting quality.

## 1. Introduction

### 1.1. Selenium in Animals and Humans

Selenium (Se) is an essential component of selenoaminoacids and selenoproteins. It thus has multiple roles in the growth and functioning of living cells and has many crucial biological functions in animals and humans [1,2]. Selenium is also a cofactor of the enzyme glutathione peroxidase, and a catalyzer of the reduction of peroxides, which can damage cells and tissues [3]. It is thus involved in antioxidant defense [4]. As a component of iodothyronine deiodonase and thioredoxin reductase [5,6], Se is involved in the formation of thyroid hormones. Selenium plays a role in DNA synthesis, fertility, reproduction, and in muscle function by improving endurance and recovery, and slowing the ageing process [7,8]. Se also helps prevent certain cancers and reduces the incidence of viral infections, cardiovascular damage, arthritis, and altered immunological functions [9,10].

Se is biologically active at low concentrations for normal growth and development, and at moderate concentrations for homeostatic function. However, at high concentrations, Se can induce toxicity [11]. The margin between the nutritional requirement and toxicity is quite small, and outside this range, deficiency or toxicity can occur. The Food and Nutrition Board of the Institute of Medicine (USA) has proposed a Recommended Dietary Allowance (RDA) of 55 µg Se day^−1^ for adults and a tolerable upper intake of 400 µg Se day^−1^ [12].

Sub-optimal Se intake and status are correlated with a wide variety of human diseases, such as heart diseases, cystic fibrosis, cognitive decline, Alzheimer's, cancer, impairment in immune function, oxidative stress-related disorders, reduced fertility and hypothyroidism [10,13]. A severe Se deficiency, caused by a Se intake lower than 10 µg Se day^−1^, may be associated with a cardiomyopathy called Keshan disease, and with an endemic degenerative osteoarthritis known as Kashin-Beck disease [1].

On the other hand, the short-term ingestion of high levels of Se can cause nausea, vomiting and diarrhea. If the excessive consumption is chronic, it may lead to a specific disease called selenosis, and also damage the cardiovascular, gastrointestinal, neurological and hematopoietic systems [14,15,16]. Selenosis has been observed in populations exposed to high levels of dietary selenium and is associated with the consumption of high-Se crops grown on seleniferous soils. Symptoms of selenosis include hair loss, brittle hair, thickened and stratified nails, gastrointestinal disturbances, and garlic odor from breath and skin. The USA Environmental Protection Agency estimated the average dietary intake at which clinical selenosis appears as 1262 µg Se day^−1^.

### 1.2. Selenium in Plants 

There is no definitive evidence regarding whether selenium is essential for vascular plants However, it has been hypothesized that it may have beneficial biological functions in species that are able to accumulate high amounts of Se and that need Se for their normal growth [17,18,19]. The accumulation of selenium in plants varies in relation to plant species, and is affected by soil Se concentration, soil properties and the chemical form of Se [20,21].

The leaf Se concentration in most plants, defined as non-accumulators, is usually below 100 mg Se kg^−1^ dry weight. Only a selected number of plant species grown in areas rich in selenium can accumulate a high amount of selenium in the leaves. These plants are classified as hyperaccumulators when they accumulate more than 1000 mg kg^−1^ dry weight (*Astralagus* and *Stanleya pinnata*), and indicators or secondary accumulators when they accumulate 100–1000 mg kg^−1^ dry weight (*Brassica juncea*, *Melilotus*, *Atriplex*) [22].

Most agricultural crops have a much lower tolerance (<50 mg Se kg^−1^), however Brassicaceae, onion, garlic and some mushrooms have a high Se concentration due to their high content of sulfur compounds. Selenium has a close similarity in terms of properties to sulfur and it can play the same role as S in biochemical systems. Uptake, translocation and metabolism of Se mimics those of S, thus the substitution of sulfur with selenium results in selenium analogue compounds that increase the selenium content [18,23,24]. Legumes, especially lentils [25], contain a high Se concentration, however nuts rich in proteins, such as pistachios, walnuts and Brazil nuts, have been found to have the richest selenium content. Fruits usually contain a low selenium content, probably due to the low protein and high-water content [26].

At appropriate concentrations, Se positively affects seed germination and plant growth [17,27,28,29]. Se protects plants from several abiotic stresses, including ultraviolet light, heavy metals and arsenic, and biotic stress, including pathogens and herbivores [30]. Se counteracts oxidative stress by inhibiting lipid peroxidation [19,31], and increases gluthatione peroxidase (GSH-Px) activity [27,32,33]. The enhanced antioxidation associated with an increase in glutathione peroxidase activity may delay plant senescence and decrease postharvest losses.

At high concentrations, Se acts as a pro-oxidant, inhibiting the growth and germination of seeds and reducing yields [17,27,34].

Selenium can affect the quality of vegetables and fruit. An increased cellular content of linoleic acid and sterols and a decreased oleic acid content have been observed in *Camelia oleifera* plants treated with selenium [35]. Se treatment had a positive effect on maintaining the sensory and the postharvest quality by reducing the respiratory intensity and ethylene production in broccoli [36], by decreasing phenylalanine ammonia-lyase (PAL) activity and ethylene production in lettuce and chicory [37], and by diminishing ethylene production in tomatoes [38,39]. In green tea, Se increased plant yield, total amino acid, and vitamin C content [28]. In peaches and pears, Se spraying of the canopy slowed down the rate of fruit softening, and thus increased the shelf-life [40]. The application of selenium may be effective in controlling postharvest gray mold disease in tomato fruits caused by *Botritis cinerea* [41].

Due to its ability to increase the antioxidant defense of plants [18,42,43], Se has been found to delay plant senescence [17,27,37] and fruit ripening [39,40,44,45] in several horticultural species, which could lead to decreased postharvest loss. The antioxidant capacity of Se and improved gluthatione peroxidase activity are related [17,19,27], which suggests the presence of a Se-dependent GSH-Px [17].

## 2. Selenium Enrichment of Agricultural Crops

Several strategies can improve a suboptimal selenium status, including a diversified diet, food supplements, fortification of foodstuffs, and the biofortification of plants [46,47,48,49,50].

A diversified diet can provide a good intake of minerals, proteins and vitamins, however in many socio-economic contexts around the world, access to diverse diets is not possible [50,51,52,53,54].

Se supplements include sodium selenate and sodium selenite (inorganic forms), and selenium-enriched yeast, selenomethionine and selenocysteine (organic forms). However, since 2002 in the EU only inorganic forms of selenium are permissible as food supplements. Se-enriched yeast appears to have a high variability with respect to its Se content and speciation, but represents a good way to increase the consumption of Se by humans [46].

The addition of nutrients, such as minerals and vitamins, to increase the nutritional quality of processed food is called fortification [55,56]. It is practiced to restore nutrients lost during food processing or in areas characterized by problems of malnutrition and aimed at correcting deficiencies in one or more nutrients.

Increasing the concentration of micronutrient in plants in order to improve the nutritional quality of plant-based food during plant growth rather than during crop processing is known as biofortification [57,58,59,60].

The ability of some crops to accumulate selenium is crucial for human nutrition and health. Plants are the first link in the food chain which ends with humans. Increasing the Se content in plant products, including leafy and fruity vegetables, fruit crops, and cereals, without exceeding the toxic threshold, is thus a good way to increase animal and human Se intake and may have positive effects on long-term health.

The main strategies to increase both mineral levels and their bioavailability in the edible part of staple crops include agronomic intervention and plant breeding.

Agronomic biofortification is performed through the application of mineral elements with a good mobility, such as I, Zn, and Se, in the soil and in the plants [58,61,62,63]. This strategy has mainly been conducted in Northern and Central Europe and at a national scale was adopted in Finland in 1984, where Se was added to all agricultural fertilizers because of the very low consumption of Se by Finnish people. The program was successful in increasing Se concentration in foodstuffs and Se intake in humans [48,64]. Se biofortification programs under Mediterranean conditions may include legumes, which constitute the main source of dietary proteins for a large share of the world's population and can accumulate a higher amount of Se in the grain compared to cereals. In peas, the application of a small quantity of sodium selenate resulted in a great increase in Se concentration in the grain [65].

Se biofortification of fruits, vegetables and cereals is a good way to increase the supplementation of selenium by humans and has been reviewed elsewhere [30,48,62,66,67,68]. In many Se-enriched plants most Se is in the major organic form (selenomethionine, selenocysteine and methylselenocysteine). It is more available to humans and more efficient in increasing the selenium content, especially in the blood, than the inorganic forms. The consumption of Se-enriched plants thus appears to be beneficial [47,48,69,70]. For example, in onions, garlic and broccoli, Se is mostly present as Se-methylselenocysteine or γ-glutamyl-Se-methylselenocysteine, with some differences according to the plant species and application doses. In Se-enriched onions the main Se chemical form is γ-glutamyl-Se-methylselenocysteine (about 63% of total Se), followed by selenate (10%), and selenomethionine (5%) [71,72]. γ-glutamyl-Se-methylselenocysteine is the predominant Se species (73%) which can also be found in enriched garlic. Other chemical species are present at lower concentrations: seleniomethinine (13%), γ-glutamyl-selenomethionine (4%), Se-methylselenocysteine (3%) and selenate (2%) [73]. In Se-enriched broccoli Se mostly consists of Se-methylselenocysteine (45%) and small amounts of selenomethionine and selenate [74].

The potential of Se fortification of crops by genetic manipulation is still not clear. Some evidence indicates that Se content can be increased in grains [59,63,64,67] and lentils [25] by breading, thus providing an alternative to agronomic fortification and minimizing the use of Se fertilizers, however further efforts are needed.

Possible negative effects of selenium enrichment to plants are mainly caused by interference with sulfur metabolism, since Se is incorporated into organic compounds which can act as Se analogues (selenocysteine and selenomethionine) of essential S compounds (cysteine and methionine) [18].

The amount and the chemical form of Se found in natural products is well known, and the main foods providing selenium in the diet are nuts, bread, cereals, meat, fish, eggs, and milk/dairy products, as reviewed by Fairweather-Tait et al. [50].

The accumulation of selenium in food crops induced by biofortification must satisfy a rational approach to selenium supplementation according to the recommended RDA, without leading to Se intoxication and without producing phytotoxic effects or reducing the agricultural production.

### Selenium Supplementation in Plants

There are four main methods for enriching plants with selenium: (1) adding Se to the soil; (2) soaking seeds in a Se solution before sowing; (3) foliar or fruit spraying; and (4) hydroponic cultivation with a nutrient solution containing Se.

The addition of Se fertilizers to the soil is an appropriate way to biofortify high amounts of foodstuffs and to increase the Se content in soils that have a low selenium content [61,75,76,77,78]. However, high amounts of Se need to be applied to the soil to obtain Se plant concentrations equal to other fertilization methods [49,79]. The soil selenium content can be increased by fertilizing soil with salt (selenate or selenite) [61] or by the incorporation of Se-hyperaccumulator plants in the soil. Good results in the biofortification of carrots and broccoli have been obtained by adding Se-enriched *Stanleya pinnata* plants to soil [80]. As a Se-hyperaccumulator, *Stanleya pinnata* can be used for the phytoremediation of soil with a high concentration of Se [81], and the Se-enriched plant material can then be incorporated into the soil thus combining phytoremediation and biofortification [82].

Selenium spraying has been used to enhance the Se content in potatoes [83], rice [84], soybeans [85], buckwheat and pumpkins [86], garlic [87], carrots [88], broccoli [89], cabbages [76], radishes [90], basil [91,92,93,94], tomatoes [44,95], peaches and pears [40], and grapes [96]. Results of spraying depend on the characteristics of the leaf and fruit surface, such as the presence of hairs, the characteristics of the epicarp, the chemical composition of the epicuticular wax, or the deposition of wax platelets [40]. Foliar application of Se has proven effective for the biofortification of *Chicorium intybus* L. plants: Se concentrations in sprayed plants [97] were found to be higher compared with plants grown in a nutrient solution containing Se [37,98]. In addition, foliar spraying is preferable to soil application due to the lower amount of Se generally used, and because no residual effects have been observed. Foliar application involves a minimum consumption of Se salts and is an effective, safe and economically acceptable way of improving Se content in crops [99].

The results of biofortifying plants by soaking seeds in a solution containing Se are still not well known, however good results have been obtained in grains. Ožbolt et al. [100] found an increased Se content in buckwheat plants without any decrease in production, and an improvement in drought tolerance was found by Nawaz et al. [101] in wheat. However, Se concentrations detected in these biofortified plants were lower compared to the other methods.

Hydroponic culture enriched with selenium is useful for providing Se-enriched vegetables. Studies have been conducted on lettuce [27,34,37,102,103], sweet basil [91], chicory [37], spinach [104], chard [105] and tomatoes [34,39]. The use of a floating system makes it possible to control the concentration of Se in the growth medium and to easily adapt the Se supply to the growth stage of plants, thus avoiding salt loss [37].

In general, the supplementation of Se by foliar application or in the growth medium increases Se concentration in plant tissues without a loss of production or qualitative characteristics.

## 3. Selenium Enrichment of Leafy Vegetable: Effects on Yield, Quality and Senescence

Se biofortification of leafy vegetables has been widely studied. Plants have been grown on selenium-enriched substrates [106,107,108,109], in a nutrient solution with selenium added [37,98,104,105,110,111,112,113,114,115,116] or treated with Se foliar application [91,92,93,94,97,117]. Table 1 summarizes the various studies on the Se enrichment of leafy vegetables.

The addition of selenium has been found to significantly increase the Se content in plants, in general without negatively affecting the biomass and the quality of leaves, as in spinach [97,104,110,115,116], lettuce [102,106,107,108], basil [91,94], and chicory [37]. The addition of low doses of Se, of approximately 1.5 mg Se L^−1^ in the nutrient solution [37,102,104,108,110,115,116], or 1 mg Se L^−1^ by foliar fertilization [97], were not found to induce toxic effects in chicory plants. Similarly, foliar fertilization with 50 mg Se L^−1^ [91] or 25 mg Se m^−2^ [94] did not negatively affect the biomass of sweet basil plants. Se could also have positive effects on plant growth, increasing yield in basil [91,117], chicory and lettuce [37,98,102,116]. Rios et al. [113] observed a positive effect of Se on plant growth at application rates of 5–20 µmol Se L^−1^, no effects at 40 µmol Se L^−1^ and a reduced production of biomass at an application rate of 120 µmol Se L^−1^ [111,113,114,118].

The chemical form of Se applied influences the toxicity in plants. In general, selenate is less toxic than selenite, and biomass production is higher when selenate is supplied [102,111,113,114,115,118]. In addition, selenite is toxic for plants at lower concentrations compared to selenate [102].

An antioxidant effect of Se has been detected in Se-enriched leafy vegetables due to the increase in the antioxidant enzyme activity: lypoxigenase (LOX) [113], superoxide dismutase (SOD) [102,111], catalase (CAT) [102], ascorbate peroxidase (APX), and glutathione peroxidase (GSH-Px) [111]. An improved oxidative state was also detected by measuring the content of some oxidative markers and compounds, such as malondialdehyde (MAD), 2,2-diphenyl-1-picrylhydrazyl (DPPH), H_2_O_2_, phenols and flavonoids, and by measuring the antioxidant activity by the Ferric Reducing Antioxidant Power (FRAP) assay. A reduction in DPPH, H_2_O_2_ [116], and MDA content [91], as well as an increase in antioxidant capacity (measured by the FRAP assay), phenols [91,106,113,115,117], and flavonoid content, were detected in plants treated with low concentrations of selenate [113].

The reduced glutathione content is also an indicator of the oxidative status of plant tissues, and Se increases the content of this compound [117]. However, sometimes Se does not induce changes in phenolic compound content [93,94], but it may have a positive effect on the anthocyanin content [91]. Due to its antioxidant action, Se may have a positive effect at low concentrations thus increasing plant growth. In contrast, at high concentrations Se, especially as selenite, can act as a pro-oxidant inducing oxidative stress in leafy vegetables, by increasing H_2_O_2_ [111], MDA [113,116] content and lipid peroxidation [118].

The effect of Se on photosyntethic pigments is not clear. In general, low concentrations of Se have not been found to affect the photosynthetic pigment content in basil (treated with 50 mg Se L^−1^ or lower) [91], lettuce (treated with 0.316 mg Se L^−1^ selenite, 2.37 mg Se L^−1^ selenate or lower) [116,118], and chicory (1 mg Se L^−1^ or lower) [37]. On the other hand, higher concentrations of Se were found to decrease the content of photosynthetic pigments both in lettuce (treated with 0.474 mg Se L^−1^ selenite, 3.16 mg Se L^−1^ selenate or higher [116], 40 µg Se plant^−1^ selenite, selenium urea or imidoselenocarbamate [106]) and basil (30 mg Se L^−1^ or higher) [117].

An increase in carotenoids in basil (treated with concentrations from 30 to 120 mg Se L^−1^) [117], and of chlorophylls in spinach (treated with 1 mg Se L^−1^) [115] have also been detected.

The effect of Se on nitrogen metabolism is not clear. Ríos et al. [118] found a reduced nitrate content in Se-treated lettuce and basil plants, respectively, whereas no effects were detected in chicory [37], spinach [104], and chard [105].

A longer shelf life and preserved quality, in association with lower rates of ethylene biosynthesis have been observed in lettuce and chicory [37,98]. In addition, in senescing lettuce plants, Se has been found to increase stress tolerance by preventing the decrease in tocopherol concentration and by increasing the activity of superoxide dismutase (SOD) [27].

Table 2 summarizes the main effects induced by selenium in leafy vegetables.

## 4. Selenium Enrichment of Fruit Crops: Effects on Yield, Quality and Senescence 

Due to the antioxidant capacity of Se and its influence on ethylene biosynthesis, several experiments have been conducted to study the effects of Se biofortification on fruit quality and postharvest. In tomato (*Solanum lycopersicon* L.) selenium, as sodium selenate, has been added to nutrient solutions [39,41,45,95,109,119,120] or given to plants by foliar application [121]. Only a few studies have been conducted on the Se-enrichment of fruit trees. Peaches (*Prunus persica* Batch) and pears (*Pyrus communis* L.) [40] were sprayed with selenate solution, table grapes (*Vitis vinifera* L.) [96] were sprayed with organic selenium, and pears (*Pyrus bretschneideri* cv. ‘Huangguan’), grapes (*Vitis vinifera*, cv. ‘Kyoho’), and peaches (*Prunus persica*, cv. ‘Jinliuzaohong’) were sprayed with amino acid-chelated selenium solution.

Table 3 summarizes the various studies on the Se enrichment of fruit crops.

The addition of Se has been found to increase the Se concentration in the fruits and leaves of the treated plants of both tomato [39,41,45,95,109,119,120] and fruit trees [40,96], without affecting the yield.

Evidence of the positive effects of Se on the quality parameters of tomato fruit has been reported, such as soluble solid content [44,45,119], titrable acidity [44,119], glucose, fructose, and total sugar content [119], and firmness [44]. Se also positively affects the soluble solid content in peach and pear [40], glucose, fructose, organic acid and protein contents in grape [96]. In addition, during storage, Se delayed the decline of firmness [40,44] and titrable acidity, as well as weight loss in tomato fruits [44]. In tomato fruits, Se may also induce an increased content of pigments and of antioxidant compounds [39,45,95,120,121].

An increased net photosynthetic rate and a decreased stomatal conductance and transpiration rate were detected in pears, grapes, and peach foliar sprayed with amino acid-chelated selenium [122].

Treatments with Se showed a positive effect in improving the oxidative status of fruit and in delaying tomato fruit ripening, thus positively affecting the post-harvest shelf-life of fruit without negatively affecting the quality [44,45,120]. Se may increase the antioxidant enzyme activity [45,109] and decrease various reactive oxygen species during storage [45]. Experiments conducted by Pezzarossa et al. [39] found a delay in the onset of fruit ripening in Se-enriched tomato, which also showed a reduced rate in color change, and an earlier harvesting of control plants compared to Se-treated plants. The delay in fruit ripening may depend on a reduced ethylene production. This reduction could be due to the higher cellular concentration of selenomethionine (Se-Met) than methionine (Met), which is a precursor of ethylene in the ethylene biosynthesis pathway [23,123]. Another hypothesis regards the genes of the enzymes involved in the ethylene biosynthesis pathway. In this pathway, the conversion of S-adenosyl methionine to 1-Aminocyclopropane-1-carboxylic acid (ACC) is catalyzed by the enzyme ACC synthase (ACS). The ACC is converted into ethylene by ACC oxidase (ACO) [123]. Four genes belong to the ACO gene family; amongst them, *ACO1*, is predominantly expressed in tomato fruit [124], while *ACS2* and *ACS4*, primarily govern the ethylene production of system 2 during ripening [125]. Zhu et al. [45] found that Se can suppress the transcription of *ACO1*, *ACS2* and *ACS4*, thus reducing ethylene production in tomato fruits.

Se may have an indirect positive effect on the post-harvest storage of vegetables by reducing germination and the mycelial growth of some harmful fungi such as *Botrytis cinerea* [41,44] and *Pennicillium expansum* [126]. *Botrytis cinerea* causes gray mold decay, one of the main pre- and post-harvest diseases in fruit and vegetables [127,128], leading to economic losses [129,130]. Se also counteracts infections by *Alternari brassicola* and *Fusarium* sp. and enhances resistance to fungal diseases [131], for example reducing the damage due to *Fusarium* wilt infection in tomato [132]. In addition, high Se concentrations in plant tissues may have a positive effect by reducing invertebrate herbivory damage [131]. Table 4 summarizes the main effects induced by selenium in fruit crops.

In conclusion, biofortified food with selenium can address Se deficiencies in humans, thus increasing the amount of selenium in the diet and preventing the risks of excess Se intake which mineral supplements can induce. In horticultural crops, selenium has potential benefits in terms of storage and shelf-life. In fruit, Se may modulate the ripening process, probably through its antioxidant and anti-senescence properties with beneficial effects in terms of post-harvest commercial life, and greater benefits for human health. Further studies are needed in order to fully understand the molecular and biochemical mechanisms directly or indirectly affected by selenium in fruit tissues at ripening and during the postharvest phase.

## Figures and Tables

**Table 1:** Selenium (Se) accumulation in the edible parts of leafy vegetables in relation to the concentration, the chemical form of Se supplemented to plants, and the method of Se supplementation.

Plant Species	[Se] Supplemented	Se Chemical Form	Se Supplementation Methods	[Se] In the Edible Part	Reference
*Lactuca sativa* L. var. Acephala	0.5 and 1 mg Se L^−1^	selenate	Enrichment of nutrient solution	26 mg kg^−1^ dry weight (DW)	Diaz et al. [98]
*Lactuca sativa* L. var. Acephala	1.5 and 5 mg Se kg^−1^	selenite	Soil fertilization	20 mg kg^−1^ DW	Pezzarossa et al. [108]
*Lactuca sativa* L.	5 mg Se kg ^−1^	selenate	Soil fertilization	170 mg kg^−1^ DW	Pezzarossa et al. [108]
*Lactuca sativa* L. var. Acephala	0.5 and 1 mg Se L^−1^	selenate	Enrichment of nutrient solution	26 mg kg^−1^ DW	Malorgio et al. [37]
*Lactuca sativa*	0.16 to 5.12 mg Se L^−1^	selenate	Enrichment of nutrient solution	10 to 43.3 DW	Rios et al.; Malorgio et al.; Ramos et al.; Hawrylak-Nowak [37,102,111,113,114,116,118]
*Lactuca sativa* L. cv. Justyna	from 0.16 to 2.4 mg Se L^−1^	selenite	Nutrient solution	30.6 mg kg^−1^ DW	Hawrylak-Nowak [116]
*Lactuca sativa* L.	1 to 1000 mg Se kg^−1^	selenate	Peat fertilization	219 mg kg^−1^ DW	Businelli et al. [109]
*Lactuca sativa* L. cv. Capitata	40 µg plant^−1^	selenite, selenium urea, imidoselenocarbamate	Vermiculite-sand-peat fertilization	68.4 to 413.5 µg kg^−1^ DW	Goicoechea et al.; Sanmartín et al. [106,107]
*Chicorium intybus* L.	0.5 and 1 mg Se L^−1^	selenate	Enrichment of nutrient solution	30 mg kg^−1^ DW	Diaz et al. [98]
*Chicorium intybus* L.	1 mg Se L^−1^	selenate	Foliar fertilization	45 mg kg^−1^ DW	Germ et al. [97]
*Chicorium intybus* L.	0.5 and 1 mg Se L^−1^	selenate	Enrichment of nutrient solution	29.1 mg kg^−1^ DW	Malorgio et al. [37]
*Ocimum basilicum* L.	1 to 50 mg Se L^−1^	selenate	Foliar fertilization	7.86 to 150 mg kg^−1^ DW	Hawrylak-Nowak; Kopsell et al.; Barátová et al. [91,92,93]
*Ocimum basilicum* L.	25 and 50 mg m^−2^	selenate	Foliar fertilization	7.86 mg kg^−1^ DW	Mezeyová et al. [94]
*Ocimum basilicum* L.	2 to 32 mg Se L^−1^	selenite	Foliar application	41.5 mg kg^−1^ DW	Kopsell et al. [92]
*Spinacia oleracea* L.	0.8 and 1.6 mg Se L^−1^	selenate	Enrichment of nutrient solution	12 mg kg^−1^ fresh weight (FW)	Zhu et al. [110]
*Spinacia oleracea* L.	0.2 to 0.4 mg Se L^−1^	selenate	Enrichment of nutrient solution	15.5 mg kg^−1^ DW	Ferrarese et al. [104]
*Spinacia oleracea* L.	1 to 10 mg Se L^−1^	selenate	Enrichment of nutrient solution	3.89 mg g^−1^ DW	Saffaryazdi et al. [115]
*Beta vulgaris subsp. Vulgaris* L.	10 and 20 mg Se L^−1^	selenate	Enrichment of nutrient solution	1393 µg of Se per shoot	Hernández-Castro et al. [105]

**Table 2:** Main effects of selenium (Se) treatments on leafy vegetables.

Plant Species	Biomass	Oxidative Markers	Antioxidant Enzymes	Antioxidant Compounds	Photosynthetic Pigments	Nitrate Content	Ethylene Production	Reference
*Ocimum basilicum* L.	none			none				Barátová et al. [93]
*Lactuca sativa* L.	decrease at high [Se]							Businelli et al. [109]
*Lactuca sativa* L. var. Acephala	none						decrease	Diaz et al. [98]
*Chicorium intybus* L.	none						decrease	Diaz et al. [98]
*Spinacia oleracea* L.	none					none		Ferrarese et al. [104]
*Ocimum basilicum* L.	none	improve		increase	none			Hawrylak-Nowak [91]
*Lactuca sativa* L. cv. Justyna	none	improve			decrease at high [Se]			Hawrylak-Nowak [116]
*Beta vulgaris subsp. Vulgaris* L.	not determined (n.d).					none		Hernández-Castro et al. [105]
*Chicorium intybus* L.	none						decrease	Malorgio et al. [37]
*Lactuca sativa* L. var. Acephala	none				none	none	decrease	Malorgio et al. [37]
*Ocimum basilicum* L.	none			none				Mezeyová et al. [94]
*Ocimum basilicum* L.	n.d.			increase	decrease at high [Se]			Oraghi Ardebili et al. [117]
*Lactuca sativa* L. var. Acephala	none							Pezzarossa et al. [108]
*Lactuca sativa* L. cv. Vera	none		increase					Ramos et al. [102]
*Lactuca sativa* L. cv. Philipus	decrease at high [Se]		increase	increase				Ríos et al. [113]
*Lactuca sativa* L. cv. Philipus	decrease at high [Se]		increase					Ríos et al. [111]
*Lactuca sativa* L. cv. Philipus	decrease at high [Se]					decrease		Ríos et al. [118]
*Lactuca sativa* L. cv. Capitata	none	none or decrease			decrease			Goicoechea et al.; Sanmartín et al. [106,107]
*Spinacia oleracea* L.	none			increase	increase			Saffaryazdi et al. [115]
*Spinacia oleracea* L.	none							Zhu et al. [110]

**Table 3:** Selenium (Se) accumulation in fruit in relation to the concentration, the chemical form of Se supplemented to plants, and the method of Se supplementation.

Plant Species	[Se] Supplemented	Se Chemical Form	Se Supplementation Method	[Se] in Fruit	Reference
*Solanum lycopersicum* L.	0.5, 1, 2 mg Se L^−1^	Selenate	Enrichment of nutrients solution	5 mg kg^−1^ FW	Lee et al. [119]
*Solanum lycopersicum* L.	0.5, 1 mg Se L^−1^	Selenate	Enrichment of nutrients solution	11 mg kg^−1^ DW	Pezzarossa et al. [120]
*Solanum lycopersicum* L.	2 and 20 mg Se plant^−1^	Selenate	Foliar fertilization	4 mg kg^−1^ DW	Schiavon et al. [95]
*Solanum lycopersicum* L.	1 mg Se L^−1^	Selenate	Enrichment of nutrients solution	11.5 mg kg^−1^ DW	Pezzarossa et al. [39]
*Solanum lycopersicum* L.	1 to 30 mg Se kg^−1^ DW	Selenate	Peat fertilization	201 µg kg^−1^ FW	Businelli et al. [109]
*Solanum lycopersicum* L.	1 mg Se L^−1^	Selenate	Foliar fertilization	500 µg kg^−1^ DW	Zhu et al. [41]
*Solanum lycopersicum* L.	1 mg Se L^−1^	Selenate	Foliar fertilization	n.d.	Zhu et al. [45]
*Solanum lycopersicum* L.	150 to 300 g ha^−1^	Selenate	Foliar fertilization	n.d.	Andrejiová et al. [121]
*Prunus persica* Batch.	0 to 1 mg Se L^−1^	Selenate	Foliar and/or fruit fertilization	75 µg kg^−1^ DW	Pezzarossa et al. [40]
*Pyrus communis* L.	0 to 1 mg Se L^−1^	Selenate	Foliar and/or fruit fertilization	200 µg kg^−1^ DW	Pezzarossa et al. [40]
*Vitis vinifera* L.	120 mg L^−1^	Organic	Foliar fertilization	25 µg kg^−1^FW	Zhu et al. [96]

**Table 4:** Main effects of selenium (Se) treatments on fruit crops.

Plant Species	Biomass	Reactive Oxigen Species (ROS)	Antioxidant Enzymes	Ethylene Production	Qualitative Parameters	Post-Harvest Quality	Reference
*Solanum. lycopersicum* L.	none		increase				Businelli et al. [109]
*Solanum. lycopersicum* L.	increase				increase		Lee et al. [119]
*Solanum. lycopersicum* L.	none			decrease			Pezzarossa et al. [120]
*Prunus. persica* Batch.	none				increase	increase	Pezzarossa et al. [40]
*Pyrus. communis* L.	none				increase	increase	Pezzarossa et al. [40]
*Solanum. lycopersicum* L.	none						Pezzarossa et al. [39]
*Solanum. lycopersicum* L.	none						Schiavon et al. [95]
*Solanum. lycopersicum* L.	n.d.				increase	increase	Zhu et al. [41]
*Solanum. lycopersicum* L.	n.d.	decrease	increase	decrease			Zhu et al. [45]
*Solanum. lycopersicum* L.	n.d.				increase		Andrejiová et al. [121]
*Vitis. vinifera* L.	n.d.				increase		Zhu et al. [96]
